# Species composition of three size fractions of zooplankton used in routine monitoring of the Barents Sea ecosystem

**DOI:** 10.1093/plankt/fbab056

**Published:** 2021-08-31

**Authors:** Hein Rune Skjoldal

**Affiliations:** INSTITUTE OF MARINE RESEARCH, PO Box 1870 Nordnes, N-5817 Bergen, NORWAY

**Keywords:** biomass, size fractionation, trait-based, Calanus, copepods

## Abstract

Size fractionation with 2000 and 1000 μm screens is used by the Institute of Marine Research in Norway in routine monitoring of zooplankton biomass. This study examines the separation of taxa by this procedure. For copepods and cladocerans, the fractionation separates individuals according to their size in a consistent and predictable manner. Individuals up to 0.4 mm in width are contained in the small fraction (<1 mm). From width 0.4 to 0.8 mm, there is a progressive shift from the small to the medium fraction (1–2 mm). From about 0.8 mm width, individuals start to be contained in the large fraction (>2 mm). For *Calanus finmarchicus*, young copepodites CI–CIII are contained in the small fraction, while the older stages CV and adults are contained in the medium fraction. Small copepods (*Oithona*, *Oncaea*, *Microcalanus*, *Pseudocalanus*) are contained in the small fraction, as are most appendicularians and meroplanktonic invertebrate larvae. The large fraction includes large copepods, larger individuals of chaetognaths, krill and amphipods. The consistency of separation of taxa by size will help to interpret and improve the ecological relevance of results on size-fractioned zooplankton biomass in the Barents Sea as well as other high-latitude areas.

## INTRODUCTION

Zooplankton as herbivores and omnivores provide the main link between phytoplankton primary production and planktivorous fishes and other consumers like seabirds and baleen whales in marine ecosystems (Skjoldal *et al.,* 2004). Zooplankton is commonly monitored to provide information on food and feeding conditions for pelagic planktivorous fish and to document changes in the ecosystem related to climate change and altered productivity (e.g. Beaugrand *et al.,* 2002; Edwards *et al.,* 2010; WGIBAR, 2020).

The Institute of Marine Research (IMR) in Norway applies a standard method to monitor zooplankton, which involves determination of dry weight biomass in three size fractions. Each sample from the cod-end of a plankton net is split in two halves, one for biomass and one for species composition, and biomass is determined after wet sieving through three consecutive screens with mesh size of 2000, 1000 and 180 μm, respectively. The biomass retained on these screens is denoted large (>2 mm), medium (1–2 mm) and small (<1 mm) fractions. The IMR method was described in some detail by Melle *et al.* (2004) and used as the basic method in the ICES/GLOBEC gear intercomparison workshop in 1993 (Skjoldal *et al.,* 2013). The method has been routinely used in monitoring in the Barents Sea since the mid-1980s (Skjoldal *et al.,* 1992; Dalpadado et al., 2002, 2003, 2012, 2014; Stige *et al.,* 2014) and in the Norwegian Sea since the early 1990s (Melle *et al.,* 2004).

The general experience from practical use is that the IMR method of zooplankton biomass determination appears to be robust and reproducible. It gave consistent results in the mentioned gear intercomparison exercise (Skjoldal *et al.,* 2013), and it has also revealed consistent spatial and temporal patterns in zooplankton biomass in the Barents Sea over the recent decades (Stige *et al.,* 2014; Skjoldal *et al.,* 2018). The use of size fractions is a trait-based approach since body size is known to be an important factor for which organisms are seen and eaten by visually feeding fish, as well as captured by other types of predators in the trophic interactions of the pelagic food web.

We have general qualitative information on which taxa are found in the three size fractions. Such information has been routinely noted in cruise journals from quick visual examination (usually under a stereomicroscope) of which species or groups are dominant in the fractioned samples when they are transferred to the weighing trays before they are dried. Here we report quantitative results from a study where the half-sample for taxonomic analysis was size fractioned in the same way as the biomass samples before formalin preservation. The aim is to provide a better description of how well the size fractionation separates species (including copepodite stages) and groups of zooplankton. This information is important when interpreting and evaluating recent and on-going changes in the zooplankton community in the Barents Sea. While the study is carried out with samples from the Barents Sea, the results reveal general relationships between the size of organisms and fractionation and thus have wider applicability in other marine ecosystems.

## MATERIALS AND METHODS

Samples for this study were collected during the joint Norwegian–Russian ecosystem survey in 2016 from the vessel “Eros” in the western and central Barents Sea (73–78°N, 19–38°E). Single hauls were collected with WP-2 net (0.25 m^2^ mouth area, 180 μm mesh size; Skjoldal *et al.,* 2019) from eight stations located in the Bear Island Trench and Hopen Deep and on the Spitsbergen Bank and Great Bank between 20 August and 15 September at water depths varying from 63 to 462 m (Table S-1). The stations were located in the Atlantic water domain (at latitudes 73–76°N) or in the Polar Front region for the three northernmost stations (at latitudes 77–78°N) (Melle and Skjoldal, 1998). The hauls were made from near the seafloor to the surface. The content collected in the cod-end was split in two halves with a Motoda splitter, and one half-sample was processed for routine biomass determination, which included successive wet sieving through 2000, 1000 and 180 μm plankton gauze screens (Melle *et al.,* 2004; Skjoldal *et al.,* 2013; Hassel *et al.,* 2020). In the fractionation procedure, the sample was poured into the 2000 μm sieve and washed by swirling, shaking and lifting the sieve in a tray filled with about 2 L of seawater. This seawater, containing the zooplankton that passed through the coarser sieve, was poured into the next sieve (1000 μm) and the process repeated. The seawater from washing the 1000 μm screen was finally poured into the 180 μm screen where the smaller organisms were retained.

The second half-sample, which is routinely preserved in buffered 4% formaldehyde, was put through the same procedure for size fractionation used for the biomass samples, before preservation. The contents on the three screens were transferred to plastic bottles and preserved with formaldehyde following the same routine as for the unfractionated sample. Back in the laboratory, the samples were processed according to the routine procedure for taxonomic analysis at IMR (Hassel *et al.,* 2020). This includes adaptive subsampling, dependent on the size of the sample (large or small amount of zooplankton), aimed at counting large and less frequent taxa in a large proportion of the sample (the whole half-sample for large organisms such as chaetognaths), while counting small and more abundant taxa (such as *Oithona* spp.) in a small subsample. We note that this has statistical implications. The subsampling is designed to provide a “sufficient” count of the most common and abundant taxa. In practice, this means that subsampling is guided by the need to count at least hundred individual copepodites of *Calanus* spp., which are a dominant component of mesozooplankton biomass in the Barents Sea (Aarflot *et al.,* 2018). Less abundant taxa in the same size range are therefore counted with low numbers and the associated variance becomes large due to the subsampling (Skjoldal *et al.,* 2013).

The three species of *Calanus* (*C. finmarchicus*, *C. glacialis* and *C. hyperboreus*) are counted separately for each copepodite stage CI to CVI (adult females and males). For *Metrida* spp., *Pseudo-/Paracalanus* spp. and *Pareuchaeta* spp., counts are made for CI–CIII combined, CIV–CV combined, and CVI (adults). *Pseudocalanus* is not separated from *Paracalanus* in routine analysis, but *Pseudocalanus* species are predominant in the samples from the Barents Sea where *Paracalanus* species are rear or absent (Fredrika Norrbin, Arctic University of Norway, personal communication). For other copepods, such as *Oithona* spp. and *Microcalanus pusillus*, counts are made for all copepodite stages combined. We note that for small copepods, the young copepodites are not sampled or sampled with low efficiency due to their small size (Skjoldal *et al.,* 2013). Other species (e.g. *Fritillaria borealis*) or groups (e.g. copepod nauplii or gastropod larvae) are counted as single taxa. A total of 47 taxa (including 25 copepodite stages of six species of copepods) are listed in Table S-1. A few more taxa were recorded in the material, but they were rare and amounted to only 0.03% of the sum of total counts.

The species composition of the eight samples was “typical” for the late summer–autumn season in the Barents Sea but varied much across the eight stations (Table S-1). For the examination of how the various taxa are distributed in the three size fractions, the counts for the eight stations were summed. This reduces or eliminates the effect of large uncertainty associated with low counts for single station data. For the more abundant taxa, the results for separate stations (excluding stations where the taxa were in low abundance) are used to illustrate the variability and consistency in the size separation.

For copepods, we have used information on linear size (width) of copepodite stages to examine the relationships between separation into the three fractions and size of the individuals. Size were taken from Skjoldal *et al.* (2013; their Table 3) for most copepod taxa. The size of *C. hyperboreus* and *C. glacialis* was taken from Hirche (1997) and Madsen *et al.* (2001), respectively, and width was calculated from prosome length using the same ratio (0.3) as for *C. finmarchicus* (Skjoldal *et al.,* 2013). Cladocerans are included in this analysis, using information on mean width of the taxa from Skjoldal *et al.* (2013). The data on width of the copepods and cladocerans are included in Table S-2.

## RESULTS

### Separation of taxa in the three size fractions

*Calanus finmarchicus* was the most abundant “large” copepod with a mean abundance over the eight stations of about 27 000 individuals m^−2^ (standard deviation, SD, 26 000), dominated by copepodite stages CIII–CV (Table S-1). *Calanus glacialis* and *C. hyperboreus* were less abundant, by one and two orders of magnitude compared to *C. finmarchicus*. *Metridia* spp. (mostly *M. longa*; not separated from *M. lucens* for the young copepodites) were the second most abundant “large” copepod with a mean abundance of about 13 000 individuals m^−2^ (SD 12 000). The most abundant taxon overall was the small copepod *Oithona* spp. (mostly *Oithona similis*; Norrbin, 1991) with a mean abundance of about 130 000 individuals m^−2^ (SD 65 000).

Distribution of number of individuals in the three size fractions is summarized in Table S-2 for 46 taxa. About 90% of the total number of individuals (averaged over the stations) were counted in the small size fraction, 7.6% in the medium fraction and 2.3% in the large fraction. The total number of individuals (of all taxa) is compared with dry weight biomass (from the other half-sample) for the three size fractions in Table S-3. The biomass per individual (biomass divided by total number of individuals) was about 200, 60 and 10 μg dry weight for the large, medium and small fractions, respectively.

**Fig. 1:**
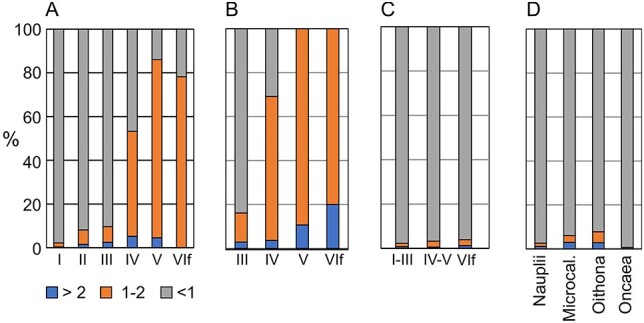
Mean percentage contribution (%) of copepod abundance in three size fractions (>2, 1–2 and <1 mm) for (**A**) copepodite stages CI–CVI of *Calanus finmarchicus*, (**B**) copepodite stages CIII–CVI of *C. glacialis*, (**C**) copepodite stages of *Pseudocalanus* spp. and (**D**) copepod nauplii, and all copepodite stages of *Microcalanus*, *Oithona* and *Oncaea* species.

*Calanus finmarchicus* showed a progressive trend of being collected less in the small size fraction and more in the medium fraction with increasing copepodite developmental stage (Fig. 1A). Stages CI–CIII were collected mostly in the small fraction (90% or more), CIV was collected about equally in the small and medium fractions, while CV and adults were collected mainly in the medium fraction. A similar trend was seen for *C. glacialis* (Fig. 1B), but with a shift towards more individuals being collected in the medium and large fractions for each of the copepodite stages CIII–CVI compared to *C. finmarchicus*. The larger *C. hyperboreus* was shifted upwards in fractions even more, with about 90% of CIV collected in the medium fraction and 30% of CV collected in the large fraction (Table S-2). For *Metrida* spp., about 90% of CI–CIII was collected in the small fraction, whereas the combined CIV–CV stages were collected about 60% in the small fraction and 40% in the medium fraction (Table S-2).

**Fig. 2:**
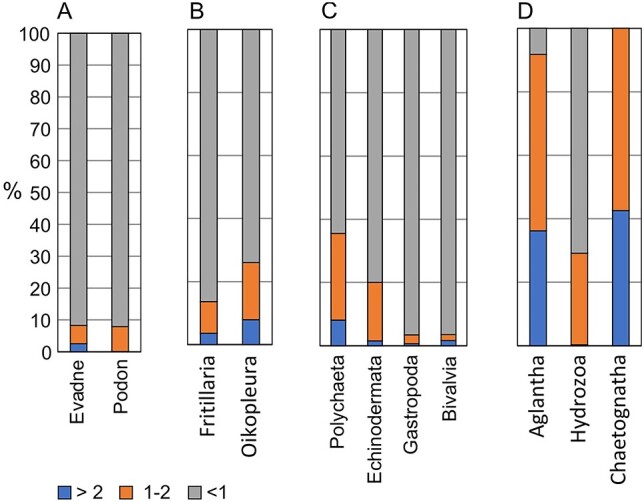
Mean percentage contribution (%) of zooplankton abundance in three size fractions (>2, 1–2 and <1 mm) for (**A**) cladocerans (*Evadne* and *Podon* species), (**B**) appendicularians (*Fritillaria borealis* and *Oikopleura* sp.), (**C**) invertebrate larvae (polychaetes, echinoderms, gastropods, and bivalves) and (**D**) the hydromedusa *Aglantha digitale*, other hydrozoans and chaetognaths (*Eukrohnia* and *Sagitta* species).

Small copepods were generally collected in the small size fraction. *Pseudocalanus* spp. were collected with 95% of the individuals in the small fraction, even for the adult stage (Fig. 1C). Similar results were found for *Oithona* spp., *Oncaea* spp., *M. pusillus* and copepod nauplii (Fig. 1D). Cladocerans (*Evadne* and *Podon* species) were also collected mainly in the small fraction (>90% of the individuals; Fig. 2A), as were bivalve and gastropod larvae (>95%, Fig. 2C). Appendicularians (*F. borealis* and *Oikopleura* spp.) and echinoderm and polychaete larvae were similarly collected with most individuals in the small fraction, but with somewhat higher proportions in the medium and large size fractions (15–35% for the two fractions combined; Fig. 2B and C). Chaetognaths and the hydrozoan medusa *Aglantha digitale* were collected mainly in the medium and large size fractions (around 60 and 40%, respectively; Fig. 2D).

The spatial consistency of the size fractionation was examined for *C. finmarchicus*, which was the most abundant copepod species with counts for all copepodite stages. Across the eight sampling stations, copepodite stage CIII was always sampled with 80% or more of the individuals in the small size fraction (Fig. S-1). Stage CIV was distributed with somewhat varying proportions (30–70%) between the small and medium fractions. Stage CV was collected with about 90% or more of the individuals in the medium fraction at all but two stations where the proportion was lower at about 50%.

### Effect of organism size on size fractionation

**Fig. 3:**
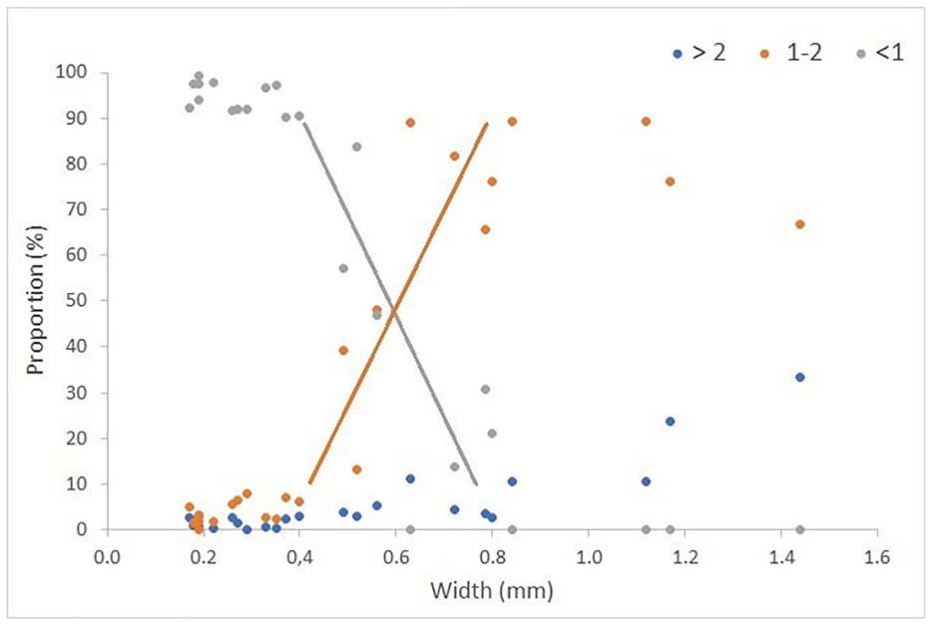
Proportional distribution (%) of biomass in three size fractions (>2, 1–2 and <1 mm) for copepods (9 species or genera including 19 separate or combined copepodite stages, plus copepod nauplii) and cladocerans (two species or genus) plotted versus size (width) of individuals. The taxa (species, genus, stage) and data are given in Table S-2. The lines are linear regressions for the segments of data between 0.4 and 0.8 mm width for the small and medium fractions.

The distribution of individuals in the three size fractions followed a consistent pattern with respect to size of the organisms, expressed as the width of copepods and cladocerans (Fig. 3). Some few individuals of the smallest organisms were collected in the large and medium fractions, with 1.5% (large) and 3% (medium) on average for organisms smaller than 0.4 mm in width (ranges from 0 up to 3% and 8%, respectively). For the size range from 0.4 to 0.8 mm, there was a progressive decrease of the proportion of individuals collected in the small fraction and a corresponding increase in the medium fraction. In this size range, the proportion occurring in the large fraction remained low (4% on average). The switch from dominance of occurrence in the small to the medium fraction followed a predictable pattern with linear regressions (for the small and medium fractions separately) giving *R*^2^ of 0.61 (*n* = 10) in both cases. Linear regressions were used to approximate the central portions of assumed underlying sigmoid-shaped, logistic equation model relationships (Nichols and Thompson, 1991). The regressions crossed with equal proportions in the two fractions at about 0.55 mm width.

For size >0.8 mm, the large fraction started to increase while the medium fraction correspondingly decreased. None of the larger organisms were collected in the small fraction. The number of data points (taxa) in the higher end of the size scale was low and prevents calculation of reliable regressions. However, the data suggest (by subjective eye-fitted extrapolation based on only three data points) that 50% collection in the large fraction would occur at a size of around 1.8 mm width (Fig. 3).

## DISCUSSION

### Taxonomic composition of the three size fractions

The size fractionation procedure used for zooplankton biomass determination has produced consistent patterns in monitoring of zooplankton biomass in the Barents Sea (Stige *et al.,* 2014; Skjoldal *et al.,* 2018) and in zooplankton sampling comparisons (Skjoldal *et al.,* 2013, 2019). The stable and reproducible nature of biomass results suggest a consistency in size fractionation at the taxonomic level, which is what the present results show. The size fractionation is strictly related to the size of zooplankton individuals, as our results demonstrate for the groups of copepods and cladocerans (see Fig. 3), which have a firm and regular body shape due to the exoskeleton.

The size dependency allows us to describe and predict the distribution of copepods in the three size fractions. Small copepods are collected in the small fraction (<1 mm). This includes the numerically dominant species of *Oithona*, *Oncaea*, *Microcalanus* and *Pseudocalanus* (including the adult stage), as well as the young copepodite stages (CI–CIII and partly CIV) of *C. finmarchicus*, *C. glacialis* and *M. longa*. The older copepodite stages (CV and adults, and partly CIV) of the *Calanus* and *Metridia* species are collected in the medium fraction (1–2 mm). For *C. finmarchicus* and *M. longa*, their size (width 0.7–0.8 mm and 0.6 mm, respectively) makes them pass though the 2 mm screen. For the larger species *C. glacialis* (width 1.0–1.1 mm), some individuals are retained in the large fraction (10–20%), but most of them are in the medium fraction (see Fig. 1B).

Due to the dominant role of *C. finmarchicus* and *C. glacialis* for the mesozooplankton biomass in the Barents Sea, and since most of the biomass build-up is associated with the later copepodite stages (Aarflot *et al.,* 2018), the medium size fraction contains much of the *Calanus* biomass and drives the mesozooplankton biomass overall. For the larger species *C. hyperboreus*, CI may appear mainly in the small fraction, CII and CIII split between the small and medium fractions, while CIV is mainly in the medium fraction. CV and adults are collected increasingly in the large fraction (>2 mm) dependent on their size. Thus, in routine monitoring at IMR, large individuals of this species are picked out from the large fraction during sample processing, and their biomass is determined separately. Older copepodite stages of the large carnivorous copepods *Pareuchaeta norvegica* and *P. glacialis* are also found in the large fraction.

For other, non-crustacean taxa, such as appendicularians and invertebrate larvae, the growth is continuous, and they occur generally with a wide range of sizes. Their shapes can also be more irregular (e.g. echinoderm larvae) compared to copepods. We did not determine the size distribution for these groups, but we assume that they are fractioned according to size like copepods are, based on results on size-dependent retention obtained with plankton nets (Skjoldal *et al.,* 2013). In the present study, appendicularians and invertebrate larvae were collected mainly in the small fraction. Based on the results (see Fig. 2), the medium size fraction would generally contain some larger individuals of appendicularians and invertebrate larvae (meroplankton) and smaller individuals of chaetognaths and hydromedusae. The large size fraction would contain larger individuals of chaetognaths and hydromedusae, as well as amphipods and krill.

The individual size of copepods and copepodite stages vary, and this variation should be taken into account when taxonomic composition of size fractions is interpreted or predicted. For *C. finmarchicus*, the length can vary by a factor of up to two among individuals of a given copepodite stage (Marshall *et al.,* 1934). The individual variation in body size is probably one reason for the variation in retention of individuals of a copepodite stage by a given screen, e.g. the variation in retention of stage CIV of *C. finmarchicus* by the 1 mm screen in this study (Fig. S-1). The average length of *C. finmarchicus* at sampling stations also varies in space and time (typically up to about 30%), e.g. a second summer generation is generally smaller in body size than the first spring generation following overwintering (Wiborg, 1954; Tande, 1982; McLaren *et al.,* 2001; Arashkevich *et al.,* 2004). Such variation can affect the species composition of size fractions, especially for taxa where the size lies in the range of transition in retention by a screen. Thus, for copepodite stage CIV of *C. finmarchicus*, which is split in being contained in the small and medium fractions (see Fig. 1A), a size change of 30% around the mean would alter the proportions in the small to medium fractions from 70:30 to 30:70, predicted from the relationships between retention and body size in Fig. 3. While this is a marked change, it would not dramatically alter the species composition of size fractions in practical applications.

### The size fractionation procedure

The wet-sieving process used in the size fractionation of the samples for zooplankton biomass determination differs from the *in situ* filtration through the gauze of a plankton net during sampling. For plankton nets, it has been shown that organisms are retained according to width rather than length, reflecting that they are oriented in the length direction as they pass through the mesh. The median (50%) retention is when body width is approximately equal to the mesh size (square side of rectangular opening), with a steep retention line (logistic model) from nearly no individuals retained to nearly all individuals retained going from width about one-third lower than mesh size to one-third larger than mesh size (Nichols and Thompson, 1991; Hays, 1994; Skjoldal *et al.,* 2013). With the 180 μm mesh nets used commonly, this means that they start collecting organisms of width about 120 μm and collect nearly all of them when they are 240 μm in width. For a 1 mm net, the same relative sizes would mean near zero to near 100% retention at widths from about 0.7 to 1.3 mm, respectively. The 1 mm screen used to retain the medium size fraction in the biomass procedure retained organisms at smaller size, with retention starting at about 0.4 mm and being near complete at about 0.8 mm (see Fig. 3). This means that the plankton organisms, notably copepods, pass through the openings of the gauze in a different manner with wet sieving compared to *in situ* filtration in a plankton net. The wet sieving involves swirling and lifting the sieve up and down in the tray used when washing the sample through the screen.

The length of a typical copepod (prosome length) is about three times its width (see Skjoldal *et al.,* 2013, Table 3). The range of width from 0.4 to 0.8 mm therefore corresponds to a range of 1.2 to 2.4 mm in prosome length. The prosome length of CV and adults of *C. finmarchicus* is around 2.4–2.5 mm (Marshall *et al.,* 1934; Wiborg, 1954; Tande, 1982; McLaren *et al.,* 2001). The equivalent spherical diameter is about half this length or about 1.2 mm (calculated from relationship between wet weight and prosome length, e.g. Tande, 1982). Our results suggest that the retention of copepods by the 1 mm screen is determined by a combination of their width and length. Copepods of 1.2 mm length (corresponding to a CIII *C. finmarchicus* of width 0.4 mm) evidently tilt over from the flat (horizontal) position and escape through the 1 mm opening of the screen. Copepods double this size (length 2.4 mm, corresponding to CV and adult *C. finmarchicus* of width 0.8 mm) are retained by the 1 mm screen, and evidently therefore, few individuals tilt over and pass through the openings of the screen oriented in the length dimension, even if the width would allow so.

With the fractionation procedure being standardized and described in a manual (Hassel *et al.,* 2020), our results demonstrate that it gives a consistent and reproducible pattern across taxa, dependent on their size. While we have not sized invertebrate larvae and other non-crustacean taxa in this study, the consistent results found by Skjoldal *et al.* (2013) for the retention of these taxa according to size by plankton nets suggest that the size plays a similar role for the fractionation used in the biomass procedure. The details of how the various non-crustacean groups are separated in the three fractions according to their size remain to be quantified.

The results for the 1 mm screen suggest that the fractionation procedure is more gentle (less forceful in squeezing flexible organisms through the openings) compared to the *in situ* screening of the plankton when samples are collected with a net. If we extrapolate these findings for the 1 mm screen to the finest mesh of 180 μm used to collect the smallest size fraction, we would expect few organisms collected with a 180-μm mesh plankton net to be washed through the 180-μm screen used in the fractionation. With retention starting and being complete from 0.4 to 0.8 mm width for the 1 mm screen, this proportionality would correspond to 0.07 and 0.14 mm for the 0.18 mm (180 μm) screen. Since the 180-μm mesh plankton net starts to collect organisms from about 120 μm in width (Skjoldal *et al.,* 2013), some small individuals could be lost by being washed through the 0.18 mm screen. However, due to their small size and low weight, this is expected to have a negligible effect on total biomass.

The 2 mm screen started to collect copepods that were around 0.8 mm in width (see Fig. 3), which is the same relative size as for the 1 mm screen (started to collect at 0.4 mm). The number of individuals was low and did not allow us to determine the fractionation by size for the 2 mm screen. If we assume the same proportionality as for the 1 mm screen, all individuals would be collected by the 2 mm screen when they are 1.6 mm in width. Thus, for organisms in the size range between 0.8 and 1.6 mm in width, there would be a progressive shift in their contribution from the medium to the large size fraction. We note that this extrapolation is based on results obtained for copepods and may not apply directly to non-crustacean taxa.

Some organisms smaller than the retention limits (0.4 and 0.8 mm for the 1 and 2 mm screens, respectively) were collected in the medium and large fractions. The contribution was low, with about 4% of the individuals of small taxa (<0.4 and <0.8 mm in width, respectively) collected in the two fractions (see Fig. 3). We interpret this to be “contamination” by small individuals retained on the 1 and 2 mm screens due to incomplete washing of the samples. Since these small organisms have disproportionally low individual weight (due to the cubed relation between weight and linear dimension), the contamination has little consequence for the biomass distribution among the three fractions (much less than the 4% on average for numbers). While cleaner samples (less contamination by small individuals) probably could be achieved by prolonging the washing process, time is limited when large numbers of samples are to be processed on routine cruises. The standardized procedure as currently used appears to be a good compromise, producing clear results that are consistent, reproducible and predictable with respect to separation of zooplankton taxa into the three fractions according to their size, as have been demonstrated here.

### Some ecological considerations and general applicability of the fractionation procedure

As mentioned, the size fractionation is a trait-based approach since body size is an important factor for production, turnover and trophodynamics involving zooplankton (e.g. Hansen *et al.,* 1994; Banse, 1995). The choice of 2 and 1 mm screens to produce three fractions (large—>2 mm, medium—1-2 mm, and small—<1 mm) was pragmatic but guided by an aim to separate the *Calanus* species from small copepods. We can now characterize and document this separation, with small copepods in the small fraction and the “large calanoid” copepods in the medium fraction (and partly also in the large fraction for large individuals of *C. hyperboreus*). Copepods are generally the dominant component of zooplankton, especially in the cold waters of polar environments where *Calanus* species play large roles (Longhurst, 1985; Conover, 1988; Falk-Petersen *et al.,* 2009). From a trophic perspective, there is an important distinction between small and large copepods in that the former are generally too small to be seen and eaten by planktivorous fish, whereas the large copepods such as *C. finmarchicus* are visible and readily preyed upon (Aksnes and Giske, 1993; Aarflot *et al.,* 2019).

An important planktivorous species such as Atlantic herring (*Clupea harengus*) has been found to take some copepodite stage CIV of *C. finmarchicus*, but it selects predominantly the older stages CV and adults (Dalpadado *et al.,* 2000; Prokopchuk and Sentyabov, 2006). Compared to herring, Atlantic mackerel (*Scomber scombrus*) can filter-feed on somewhat smaller organisms including stage CIII of *C. finmarchicus*, but also mackerel selects the older and larger copepodite stages (Prokopchuk and Sentyabov, 2006). Blue whiting (*Micromesistius poutassou*) generally selects larger zooplankton prey such as krill and amphipods, but juveniles eat copepods including older stages of *C. finmarchicus* (Langøy *et al.,* 2012). As a gadoid species, blue whiting lacks a gill lattice and therefore do not filter-feed like herring and mackerel do (Monstad, 2004). The same is the case for polar cod (*Boreogadus saida*) and Atlantic capelin (*Mallotus villosus*), which also tend to select larger prey such as krill and amphipods (Hop and Gjøsæter, 2013). Both species, especially younger fish, eat copepods, but they select strongly for the larger copepodite stages of *C. finmarchicus* and *C. glacialis* in the Barents Sea (Orlova *et al.,* 2009).

This brief review of feeding by some of the planktivorous fishes that are important in the Norwegian and Barents seas suggests that the 1 mm screen separates well between individuals in the small fraction, which are not eaten by those fish, and individuals in the medium fraction, which constitute the main diet component for some of those plankton-feeders. However, the difference in size selection among species and size groups of planktivorous fishes must be considered when monitoring results are interpreted and used in trophodynamic contexts. An obvious exception to the “rule” that planktivorous fish eats large copepods is the planktonic fish larvae that eat predominantly small copepods and other small prey. Thus, first-feeding cod larvae eat predominantly copepod nauplii before shifting gradually to young copepodites as they grow (Ellertsen *et al.,* 1989). A similar shift has been observed for herring and capelin larvae, which eat *Calanus* copepodites when they are about 3 cm in length (Pedersen and Fossheim, 2008). Small juvenile (0-group) cod and haddock also feed on copepodites of *C. finmarchicus* in the Barents Sea (Dalpadado *et al.,* 2009). We note that included in the group of small copepods are the early stages (nauplii and young copepodites) of the large calanoid copepods such as *C. finmarchicus*. This serves to illustrate the complexity of the relationships between different size groups of zooplankton and their predators in relation to recruitment of both zooplankton and fish (Skjoldal and Melle, 1989).

The species composition of zooplankton in the Barents Sea, dominated by species of *Calanus* among the large calanoids, and *Pseudocalanus*, *Oithona* and other species of small copepods, is broadly similar to that found in other boreal and subarctic marine ecosystems in the North Atlantic as well as in the North Pacific (Cooney, 1981; Huntley *et al.,* 1983; Hassel, 1986; Conover, 1988; Melle *et al.,* 2004; Eiane and Tande, 2009; Gislason *et al.,* 2009). The similarity is partly reflecting wide distributions of species (e.g. *C. finmarchicus* in the North Atlantic; Helaouët and Beaugrand, 2007) or the presence of closely related species of the same genera (e.g. *Calanus marshallae* in the Bering Sea; Smith and Vidal, 1986). The strict relationship between size of the copepods and their separation into size fractions by the screens makes our findings applicable to other high-latitude marine ecosystems beyond the Barents Sea. The results should have applicability to studies in marine and coastal environments at lower latitudes as well, in the general sense that size fractionation separates copepods and presumably other taxa in a consistent and predictable manner according to the individual size of taxa.

Size fractionation by sieving has been used in several studies of zooplankton, especially with focus on metabolic activity, turnover and trophic position revealed by stable isotopes (e.g. Lie *et al.,* 1983; Head *et al.,* 1999; Rolff, 2000; Bănaru *et al.,* 2014; Harmelin-Vivien *et al.,* 2019). Successive sieving through 2000 and 1000 μm screens has been commonly used, while some studies have also included a 500-μm screen to improve the resolution in the lower side of the size spectrum. This might be particularly relevant in neritic and warmer environments where the size distribution of the zooplankton is skewed towards smaller size.

## CONCLUSIONS

Wet sieving though 1000 and 2000 μm screens separates zooplankton individuals according to their body size in a consistent and predictable manner. With increasing body size, the 1000-μm screen starts to retain copepods of width 0.4 mm (length 1.2 mm) and retains nearly all of them at width 0.8 mm (length 2.4 mm). This provides an effective separation of “small copepods” in the small fraction and “large” calanoid copepods in the medium fraction. Small copepods include *Oithona*, *Microcalanus*, *Pseudocalanus* and young copepodite stages (CI–III) of *C. finmarchicus*, while large copepods include older stages (CV and adults) of *C. finmarchicus*, *C. glacialis* and *M. longa*. While these findings are obtained with samples from the Barents Sea, they are broadly applicable to other boreal and subarctic ecosystems due to the wide distribution of dominant species, or the presence of closely related species, in different regional large marine ecosystems.

## Supplementary Material

Supplementary_revised_120821_fbab056
