# Supplementary material for: Species composition of three size fractions of zooplankton used in routine monitoring of the Barents Sea ecosystem
Source: J Plankton Res. 2021 Aug 31;43(5):762–72. doi: 10.1093/plankt/fbab056 (PMC8461643; doi:10.1093/plankt/fbab056)
Supplement: Supplementary_revised_120821_fbab056 [file supplementary_revised_120821_fbab056.docx]

Supplementary material

*Zooplankton species composition of three size fractions used in routine monitoring of the Barents Sea ecosystem*


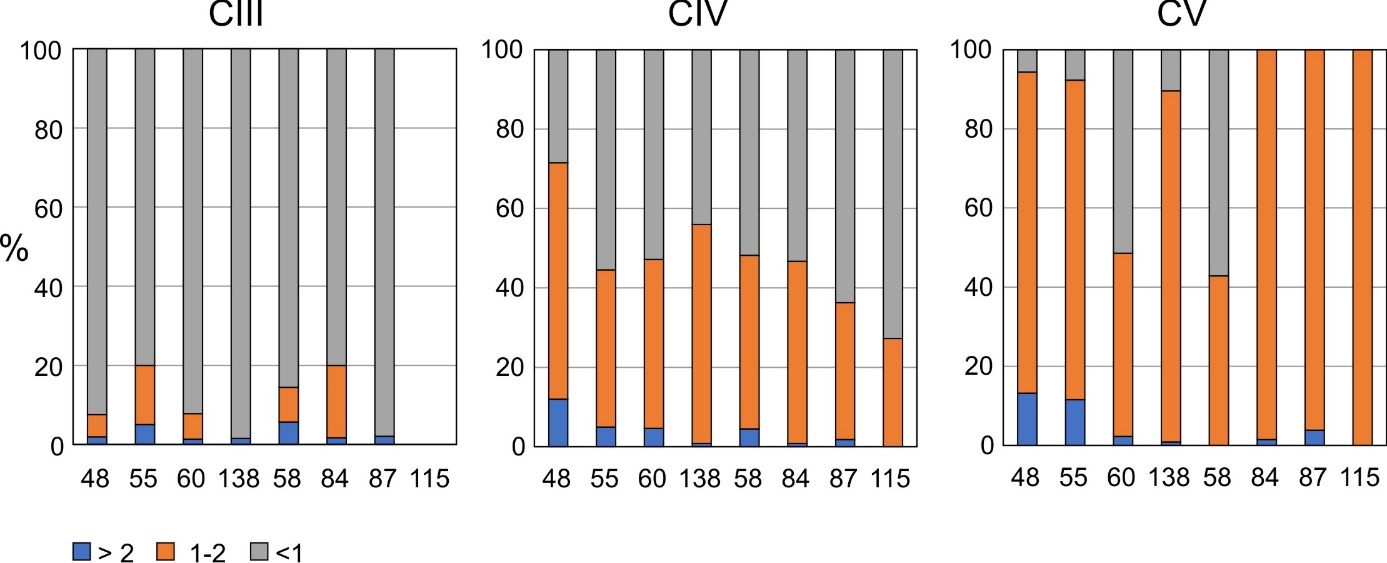


Fig. S-1 (Supplementary). Distribution (%) of number of individuals in three size fractions (>2 mm, 1-2 mm, and <1 mm) for (left) copepodite stage CIII, (middle) stage CIV, and (right) stage CV of *Calanus finmarchicus* for the eight sampling stations indicated by station numbers below the columns. Note that the sample from station 115 contained no CIII individuals.

Table S-1. Station data and abundance (number of individuals m^-2^) of identified taxa at the eight sampling stations collected from RV ‘Eros’ in August-September 2016 and used for examining the taxonomic composition of three size fractionations.

| Sampling station No. |  | 48 | 55 | 58 | 60 | 84 | 87 | 115 | 138 |
| --- | --- | --- | --- | --- | --- | --- | --- | --- | --- |
| Latitude ^o^N |  | 73.2 | 75.5 | 76.4 | 77 | 77.1 | 75 | 74 | 78 |
| Longitude ^o^E |  | 31.2 | 31.2 | 37.7 | 34 | 30.3 | 19.1 | 22 | 34 |
| Water depth (m) |  | 279 | 354 | 242 | 151 | 216 | 63 | 462 | 193 |
| Date |  | 20-Aug | 21-Aug | 22-Aug | 22-Aug | 29-Aug | 30-Aug | 9-Sep | 15-Sep |
| Species or group | Stage |  |  |  |  |  |  |  |  |
| *Calanus finmarchicus* | I | 13440 | 768 | 1728 | 8 | 32 | 512 | 0 | 0 |
|  | II | 12192 | 832 | 6016 | 792 | 0 | 256 | 0 | 256 |
|  | III | 18272 | 1920 | 10176 | 19984 | 1920 | 784 | 0 | 520 |
|  | IV | 19776 | 16160 | 7904 | 8720 | 3840 | 7232 | 704 | 16264 |
|  | V | 8992 | 3328 | 448 | 4976 | 552 | 1464 | 264 | 22208 |
|  | VI | 384 | 56 | 0 | 256 | 0 | 0 | 0 | 512 |
|  | Sum | 73056 | 23064 | 26272 | 34736 | 6344 | 10248 | 968 | 39760 |
| *Calanus glacialis* | I | 0 | 0 | 192 | 0 | 0 | 0 | 0 | 0 |
|  | II | 0 | 0 | 0 | 24 | 0 | 0 | 0 | 0 |
|  | III | 0 | 256 | 1312 | 7592 | 0 | 0 | 0 | 0 |
|  | IV | 0 | 640 | 704 | 1880 | 96 | 64 | 0 | 768 |
|  | V | 32 | 256 | 0 | 600 | 32 | 192 | 8 | 8 |
|  | VI | 64 | 0 | 0 | 16 | 0 | 0 | 0 | 0 |
|  | Sum | 96 | 1152 | 2208 | 10112 | 128 | 256 | 8 | 776 |
| *Calanus hyperboreus* | II | 0 | 0 | 64 | 0 | 0 | 0 | 0 | 0 |
|  | IV | 0 | 160 | 64 | 416 | 112 | 64 | 0 | 8 |
|  | V | 72 | 40 | 0 | 0 | 8 | 64 | 0 | 8 |
|  | VI | 8 | 0 | 0 | 0 | 8 | 0 | 0 | 16 |
|  | Sum | 80 | 200 | 128 | 416 | 128 | 128 | 0 | 32 |
| *Metridia* spp. | I-III | 0 | 25088 | 3520 | 6048 | 23712 | 4768 | 0 | 1552 |
|  | IV-V | 2464 | 8480 | 4544 | 2600 | 6304 | 11184 | 0 | 4392 |
|  | VIf | 32 | 96 | 0 | 0 | 0 | 128 | 0 | 1040 |
|  | Sum | 2496 | 33664 | 8064 | 8648 | 30016 | 16080 | 0 | 6984 |
| *Pseudo-/Paracalanus* | I-III | 32 | 4992 | 55040 | 8352 | 7040 | 12440 | 47376 | 768 |
|  | IV-V | 1600 | 6656 | 21696 | 7776 | 9056 | 19520 | 82176 | 2560 |
|  | VIf | 1024 | 0 | 0 | 0 | 64 | 56 | 5792 | 256 |
|  | Sum | 2656 | 11648 | 76736 | 16128 | 16160 | 32016 | 135344 | 3584 |
| *Paraeuchaeta* spp. | I-III | 0 | 256 | 0 | 8 | 0 | 0 | 0 | 0 |
|  | IV-V | 0 | 288 | 0 | 16 | 0 | 8 | 0 | 24 |
|  | VI | 80 | 24 | 0 | 0 | 0 | 0 | 0 | 0 |
|  | Sum | 80 | 568 | 0 | 24 | 0 | 8 | 0 | 24 |
| Copepod eggs |  | 0 | 0 | 0 | 512 | 0 | 0 | 0 | 0 |
| Copepod nauplii |  | 28288 | 2624 | 2688 | 3360 | 512 | 1024 | 3088 | 512 |
| *Acartia* sp. |  | 0 | 512 | 0 | 0 | 0 | 0 | 512 | 0 |
| *Centropages typicus* |  | 0 | 0 | 0 | 0 | 0 | 256 | 0 | 0 |
| *Microcalanus pusillus* |  | 25216 | 43360 | 14592 | 21824 | 10400 | 28408 | 16 | 13824 |
| *Oithona* spp. |  | 214976 | 149760 | 181696 | 109440 | 153568 | 167096 | 27904 | 46592 |
| *Oncaea* spp. |  | 0 | 2592 | 2048 | 512 | 2080 | 2048 | 0 | 0 |
| Amphipoda |  | 0 | 8 | 0 | 24 | 8 | 0 | 0 | 8 |
| Euphausiacea |  | 8 | 0 | 0 | 0 | 24 | 136 | 0 | 8 |
| Ostracoda |  | 0 | 0 | 0 | 0 | 0 | 0 | 512 | 8 |
| *Evadne nordmanni* |  | 2560 | 64 | 8256 | 0 | 11424 | 6168 | 0 | 0 |
| *Podon* spp. |  | 0 | 0 | 3968 | 0 | 1056 | 2760 | 0 | 0 |
| *Aglantha digitale* |  | 48 | 1128 | 528 | 40 | 952 | 208 | 0 | 208 |
| Hydrozoa |  | 0 | 2432 | 144 | 0 | 0 | 0 | 1760 | 0 |
| Polychaeta |  | 1536 | 6912 | 3584 | 128 | 552 | 0 | 0 | 0 |
|  |  |  |  |  |  |  |  |  |  |
| *Clione limacina* |  | 0 | 0 | 0 | 0 | 0 | 64 | 0 | 0 |
| Gastropoda |  | 610528 | 15040 | 42112 | 4800 | 7840 | 32800 | 3952 | 60056 |
| Bivalvia |  | 0 | 1408 | 14272 | 544 | 5056 | 12984 | 1072 | 9000 |
|  |  |  |  |  |  |  |  |  |  |
|  |  |  |  |  |  |  |  |  |  |
| Chaetognatha |  | 936 | 392 | 592 | 344 | 344 | 128 | 0 | 496 |
| Echinodermata |  | 0 | 1568 | 4096 | 3200 | 2112 | 1832 | 0 | 0 |
| *Fritillaria borealis* |  | 14528 | 7808 | 5888 | 7936 | 20096 | 5344 | 640 | 0 |
| *Oikopleura* spp. |  | 384 | 7040 | 4928 | 9696 | 5440 | 2248 | 0 | 16 |
|  |  |  |  |  |  |  |  |  |  |
| Sum taxa |  | 1055936 | 383240 | 516208 | 302488 | 327016 | 380976 | 312096 | 233048 |

Table S-2. Abundance (individuals m^-2^) averaged over 8 sampling stations for zooplankton taxa (including copepodite stages) separated in three size fractions (large >2 mm, medium 1-2 mm, and small <1 mm), and the relative distribution of individuals (%) in the three fractions. The last column gives the size as average width for copepod and cladoceran taxa.

| Species/group | Stage | No. of individuals | | | Relative (%) | | | Size |
| --- | --- | --- | --- | --- | --- | --- | --- | --- |
|  |  | Large | Medium | Small | Large | Medium | Small | (mm) |
| *Calanus finmarchicus* | I | 9 | 36 | 2016 | 0.4 | 1.7 | 97.8 | 0.22 |
|  | II | 39 | 168 | 2336 | 1.5 | 6.6 | 91.9 | 0.27 |
|  | III | 169 | 480 | 6048 | 2.5 | 7.2 | 90.3 | 0.37 |
|  | IV | 527 | 4844 | 4704 | 5.2 | 48.1 | 46.7 | 0.56 |
|  | V | 242 | 4301 | 736 | 4.6 | 81.5 | 13.9 | 0.72 |
|  | VI | 4 | 115 | 32 | 2.6 | 76.2 | 21.2 | 0.80 |
| *Calanus glacialis* | I | 0 | 24 | 0 | 0.0 | 100.0 | 0.0 | 0.31 |
|  | II | 3 | 0 | 0 | 100.0 | 0.0 | 0.0 | 0.38 |
|  | III | 33 | 152 | 960 | 2.9 | 13.3 | 83.8 | 0.52 |
|  | IV | 19 | 340 | 160 | 3.7 | 65.5 | 30.8 | 0.78 |
|  | V | 15 | 126 | 0 | 10.6 | 89.4 | 0.0 | 1.01 |
|  | VI | 2 | 8 | 0 | 20.0 | 80.0 | 0.0 | 1.12 |
| *Calanus hyperboreus* | II | 0 | 8 | 0 | 0.0 | 100.0 | 0.0 | 0.54 |
|  | IV | 11 | 92 | 0 | 10.7 | 89.3 | 0.0 | 1.12 |
|  | V | 8 | 16 | 0 | 33.3 | 66.7 | 0.0 | 1.44 |
|  | VI | 4 | 0 | 0 | 100.0 | 0.0 | 0.0 | 1.6 |
| *Metridia* spp. | I-III | 250 | 508 | 7328 | 3.1 | 6.3 | 90.6 | 0.40 |
|  | IV-V | 188 | 1960 | 2848 | 3.8 | 39.2 | 57.0 | 0.49 |
|  | VI | 18 | 144 | 0 | 11.1 | 88.9 | 0.0 | 0.63 |
| *Pseudo-/Paracalanus* | I-III | 131 | 266 | 16608 | 0.8 | 1.6 | 97.7 | 0.19 |
|  | IV-V | 104 | 504 | 18272 | 0.6 | 2.7 | 96.8 | 0.35 |
|  | VIf | 11 | 24 | 864 | 1.2 | 2.7 | 96.1 | 0.33 |
| *Pareuchaeta* spp. | I-III | 1 | 32 | 0 | 3.0 | 97.0 | 0.0 | 0.60 |
|  | IV-V | 10 | 32 | 0 | 23.8 | 76.2 | 0.0 | 1.11 |
|  | VI | 5 | 8 | 0 | 38.5 | 61.5 | 0.0 |  |
| Copepod eggs |  | 0 | 0 | 64 | 0.0 | 0.0 | 100.0 |  |
| Copepod nauplii |  | 60 | 82 | 5120 | 1.1 | 1.6 | 97.3 | 0.18 |
| *Acartia* |  | 0 | 0 | 128 | 0.0 | 0.0 | 100.0 |  |
| *Microcalanus pusillus* |  | 595 | 614 | 18496 | 3.0 | 3.1 | 93.9 | 0.19 |
| *Oithona* |  | 3787 | 6504 | 121088 | 2.9 | 5.0 | 92.2 | 0.17 |
| *Oncaea* |  | 8 | 0 | 1152 | 0.7 | 0.0 | 99.3 | 0.19 |
| Amphipoda |  | 6 | 0 | 0 | 100.0 | 0.0 | 0.0 |  |
| Euphausiacea |  | 6 | 16 | 0 | 27.3 | 72.7 | 0.0 |  |
| Ostracoda |  | 1 | 0 | 64 | 1.5 | 0.0 | 98.5 |  |
| *Evadne* |  | 91 | 204 | 3264 | 2.6 | 5.7 | 91.7 | 0.26 |
| *Podon* |  | 1 | 76 | 896 | 0.1 | 7.8 | 92.1 | 0.29 |
| *Aglantha digitale* |  | 141 | 216 | 32 | 36.2 | 55.5 | 8.2 |  |
| Hydrozoa |  | 2 | 156 | 384 | 0.4 | 28.8 | 70.8 |  |
| Polychaeta |  | 129 | 436 | 1024 | 8.1 | 27.4 | 64.4 |  |
|  |  |  |  |  |  |  |  |  |
| *Clione limacina* |  | 0 | 8 | 0 | 0.0 | 100.0 | 0.0 |  |
| Gastropoda |  | 635 | 2746 | 93760 | 0.7 | 2.8 | 96.5 |  |
| Bivalvia |  | 92 | 106 | 5344 | 1.7 | 1.9 | 96.4 |  |
|  |  |  |  |  |  |  |  |  |
|  |  |  |  |  |  |  |  |  |
| Chaetognatha |  | 172 | 232 | 0 | 42.6 | 57.4 | 0.0 |  |
| Echinodermata |  | 25 | 296 | 1280 | 1.6 | 18.5 | 80.0 |  |
| *Fritillaria borealis* |  | 280 | 780 | 6720 | 3.6 | 10.0 | 86.4 |  |
| *Oikopleura* sp. |  | 295 | 672 | 2752 | 7.9 | 18.1 | 74.0 |  |
|  |  |  |  |  |  |  |  |  |
| Sum all taxa |  | 8129 | 27332 | 324480 | 2.26 | 7.59 | 90.15 |  |

Table S-3. Biomass and number of individuals of zooplankton in three size fractions (large >2 mm, medium 1-2 mm, and small <1 mm), and biomass divided by number of individuals.

| Station | Biomass (g dry weight m-2) | | | | No. of individuals m-2 | | | Biomass/individual (µg) | | |
| --- | --- | --- | --- | --- | --- | --- | --- | --- | --- | --- |
|  | Large | Medium | Small | Total | Large | Medium | Small | Large | Medium | Small |
| 48 | 1.06 | 1.92 | 5.99 | 8.97 | 15272 | 52160 | 910848 | 70 | 37 | 7 |
| 55 | 1.96 | 2.71 | 1.74 | 6.41 | 21336 | 45496 | 246272 | 92 | 60 | 7 |
| 58 | 3.97 | 0.99 | 2.21 | 7.18 | 15736 | 26880 | 360192 | 252 | 37 | 6 |
| 60 | 0.48 | 1.78 | 1.66 | 3.92 | 3688 | 15232 | 213504 | 130 | 117 | 8 |
| 84 | 0.30 | 0.75 | 1.35 | 2.41 | 5696 | 18176 | 250368 | 53 | 41 | 5 |
| 87 | 0.27 | 0.96 | 1.72 | 2.96 | 2000 | 13312 | 306944 | 137 | 72 | 6 |
| 115 | nd | 0.12 | 1.46 | 1.58 | 0 | 5792 | 169984 | nd | 21 | 9 |
| 138 | 0.97 | 5.49 | 5.94 | 12.39 | 1400 | 42504 | 137984 | 690 | 129 | 43 |
|  |  |  |  |  |  |  |  |  |  |  |
| Mean | 1.29 | 1.84 | 2.76 | 5.73 | 8141 | 27444 | 324512 | 204 | 64 | 11 |
| SD | 1.32 | 1.68 | 1.99 | 3.72 | 8088 | 17186 | 247266 | 224 | 40 | 13 |
